# Anales del Sistema Sanitario de Navarra en 2025: Suma y sigue

**DOI:** 10.23938/ASSN.1163

**Published:** 2026-04-29

**Authors:** Pere Godoy, Carmen Beorlegui

**Affiliations:** 1 Universidad de Lleida Lleida España; 2 Institut de Recerca Biomédica [IRBLleida] Lleida Spain; 3 Centro de Investigación Biomédica en Red de Epidemiología y Salud Pública (CIBERESP) Madrid España; 4 Gobierno de Navarra Departamento de Salud Servicio de Planificación, Estrategia Sanitaria e Investigación Pamplona España

## LOGROS Y NOVEDADES

En los últimos años, la revista Anales del Sistema Sanitario de Navarra ha tenido que tomar decisiones muy relevantes para asegurar su línea editorial y garantizar su futuro. Hace cuatro años apostó por el formato de publicación electrónico y abandonó la publicación impresa, manteniendo la periodicidad cuatrimestral. Simultáneamente, para asegurar su preservación y la indexación de la revista en PubMed, se optó por el depósito en PubMed Central tras superar la consiguiente evaluación. Posteriormente, en 2023, la revista se presentó al proceso de evaluación de calidad editorial y obtuvo el sello, con mención en buenas prácticas editoriales en igualdad de género, que concede la Fundación Española de Ciencia y Tecnología (FECYT). Esta evaluación de calidad editorial se ha renovado en la convocatoria de 2025.

También solicitamos y conseguimos la inclusión de Anales del Sistema Sanitario de Navarra en el Directorio de Revistas de Acceso Abierto (DOAJ), lo que esperamos que aumente su visibilidad. Con ello también reforzamos la apuesta por el acceso abierto que ya iniciamos en 2022, con el depósito de las políticas editoriales en Dulcinea y en *Open Policy Finder*, y con la distribución de nuestros contenidos bajo licencia *Creative Commons* CC-BY SA.

Este esfuerzo ha sido reconocido en marzo de 2026 con la inclusión de Anales del Sistema Sanitario de Navarra en el *Diamond Discovery Hub*, registro europeo de publicaciones de acceso abierto diamante que cumplen con los criterios de calidad del *European Diamond Capacity Hub*, tal y como ha verificado la FECYT.

Inicialmente, el factor de impacto de la revista (JIF) en la Web of Science (Clarivate) correspondiente a 2024 disminuyó de 1,0 hasta 0,6. El descenso fue la consecuencia de considerar el contenido de la monografía “Análisis de la pandemia de COVID-19 en Navarra” como artículos de la revista. Tras cursar la oportuna reclamación, se recalculó el JIF que resultó ser, por tercer año consecutivo, 1,0. Aunque seguimos en el Q4 de la categoría *Public, Environmental & Occupational Health*, nuestra posición muestra una tendencia ascendente desde 2021 (percentil 5,17) hasta 2024 -último año evaluado- (percentil 18,4).

Como novedad, se produjo el cese de Clara Bermúdez Tamayo, nuestra Directora desde 2024, por motivos laborales. A finales de 2025 se incorporó Pere Godoy García como nuevo Director de la revista.

El objetivo de este editorial es presentar y evaluar la actividad editorial en el año 2025, estudiar sus implicaciones y valorar los desafíos editoriales que debe abordar la revista.

En los tres números del volumen 48, la revista ha publicado 48 envíos: 23 artículos originales, tres revisiones sistemáticas, un protocolo, once notas clínicas y cinco cartas al editor, además de cuatro editoriales y una colaboración especial. El porcentaje de originales coincide con el promedio de los cinco años anteriores (48%), y el número de casos clínicos ha aumentado en este volumen del 16% en 2024^1^ al 23%, reflejo de la orientación clínica de la revista. Con estas publicaciones, Anales del Sistema Sanitario de Navarra pretende mejorar el conocimiento en las diferentes áreas de los sistemas sanitarios y apoyar las intervenciones para mejorar la salud de los ciudadanos.

## INDICADORES EDITORIALES Y TENDENCIAS

A continuación, se presentan los principales datos sobre flujo de envíos, decisiones editoriales y tiempos de procesamiento de la revista Anales del Sistema Sanitario de Navarra durante el año 2025, contextualizándolos en las tendencias de años previos.

### Flujo de envíos

En 2025 se recibieron 171 envíos, cantidad similar a 2024 y por debajo de la serie histórica (>200/año).

Navarra realizó 27 envíos, un porcentaje casi idéntico al de 2024 (15,8%). La mitad de los envíos (50,9%) procedieron del resto de España y, globalmente, el 35,1% correspondió a Madrid, Andalucía, Aragón y Comunidad Valenciana, que siguen siendo las comunidades más colaboradoras y que en 2024 supusieron el 32%.

Como comentábamos en el editorial del pasado año^1^, la procedencia de más del 90% de los envíos fue nacional hasta 2019, observándose una tendencia descendente desde entonces.

Respecto a los envíos internacionales, el año 2024 fue excepcional porque se recibieron 58 envíos de China que contribuyeron enormemente a que el porcentaje de envíos de otros países llegara al 54%. En 2025 la situación se ha normalizado, con cifras que encajan en la tendencia ascendente, pero con valores inferiores a 2024. Así, los envíos de otros países han disminuido a 31,6%, por encima del 26% de 2023. El 42,6% procedía de América del Sur/Central (destacando seis envíos desde Cuba), y el 46,3% de Asia (15,3% de China).

**Figura 1:**
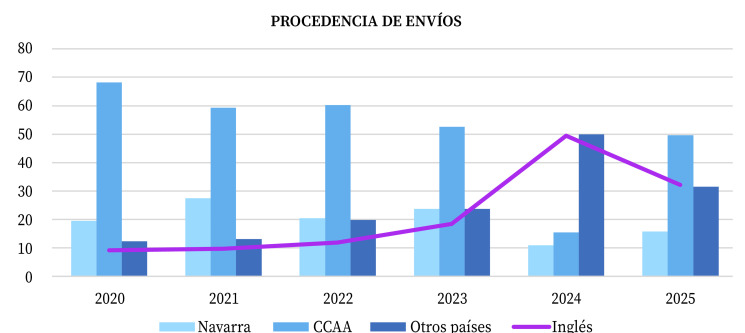
Procedencia geográfica de los envíos recibidos entre 2020 y 2025 en la revista Anales del Sistema Sanitario de Navarra, y porcentaje de los mismos en inglés.

La tendencia creciente de manuscritos redactados en inglés también se vio artefactada en 2024 por los envíos desde China; el dato de 2025 (32,2%) supone un incremento respecto al 20,4% de 2023. Todas estas tendencias se pueden apreciar en la figura 1.

Las autoras representaron el 59,7% de autorías de los envíos recibidos, y en el 62,3% de los envíos la primera autora era mujer, mejorando los porcentajes de 2024.

### Decisiones editoriales

Durante 2025 se tomaron 177 decisiones editoriales correspondientes a algunos envíos de 2024 y a la mayoría de los de 2025. Se aceptaron 42 envíos, lo que representa una tasa de aceptación del 23,7%, en la media histórica de la revista (25%).

Se rechazaron 135 envíos (76,3%). La tasa de rechazo sin revisión por pares alcanzó el 84,4% de los rechazados. Este dato supone un incremento respecto de 2024 (71%), lo que puede indicar un cribado inicial más riguroso en consonancia con el objetivo marcado en 2023 de mantener la calidad disminuyendo los tiempos de procesamiento desde las primeras etapas del proceso editorial.

La primera autoría fue femenina en el 51,5% de los envíos aceptados y en el 47,3% de los rechazados. El 50,5% de personas expertas que revisaron los envíos fueron mujeres. Estos datos cumplen con los estándares de paridad mantenidos por Anales del Sistema Sanitario de Navarra durante los últimos años y que contribuyeron a la obtención de la Mención de Buenas Prácticas Editoriales en Igualdad de Género.

### Tiempos de procesamiento

El tiempo promedio hasta el rechazo del envío fue similar a 2025 (20,1 frente a 19,1 días). Mientras que la decisión de rechazo directo se retrasó más de cinco días respecto al año anterior (14,8 frente a 8,12 días), el tiempo requerido para tomar la decisión de rechazar un envío tras pasar por revisores disminuyó en más de 12 días (55,8 frente a 68,4). Estas variaciones reflejan el esfuerzo por agilizar los tiempos del proceso editorial, pero también el impacto que circunstancias ajenas tienen en una revista con recursos limitados como Anales del Sistema Sanitario de Navarra.

Desde su recepción, los envíos aceptados tardaron cinco semanas menos en publicarse que en 2025 (20 frente a 25), y la decisión de aceptación definitiva se tomó en 95,7 días, rebajando los tiempos de años anteriores.

La tabla 1 resume las principales métricas editoriales en los seis últimos años (2020-2025), incluyendo número de envíos recibidos y aceptados, tasas de rechazo (sin revisión y tras pasar por rondas de revisión) y los tiempos promedio hasta el rechazo o la aceptación definitiva.

**Tabla 1:** Estadísticas editoriales sobre envíos aceptados y rechazados (número, porcentaje y tiempo) en los últimos cinco años

	2025	2024	2023	2022	2021	2020
**Envíos**	171	182	152	186	258	337
**Decisiones**						
N	177	174	156	191	269	307
aceptados	42	37	31	33	41	68
rechazados	135	137	125	158	228	239
rechazados (sin revisión)	111	103	80	129	187	147
**Tasa anual (%)**						
de aceptación	23,7	21,3	19,9	17,3	15,3	22,2
de rechazo sin revisión	76,3	59,2	51,2	67,4	69,5	47,9
de rechazo tras revisión	15,6	18,3	26,9	15,3	15,2	29,9
**Tiempo (días) desde recepción hasta**						
rechazo	20	18	25	23	34	50
aceptación definitiva	96	138	132	139	131	144

## AUTORÍA Y PROCEDENCIA DE LOS ARTÍCULOS PUBLICADOS EN 2025

Los 48 manuscritos publicados fueron firmados por 197 personas. El número de autorías fluctuó entre 1 y 12, con un promedio de 5,1 por manuscrito. Las editoriales, cartas a la editora y casos clínicos presentaron menos autorías. El 81% de artículos presentaban colaboración intra- o inter-institucional; el 48% de artículos estaban firmados por al menos un autor con múltiples afiliaciones.

El 61% de autores fueron mujeres, firmando el 60% de las primeras autorías y el 56% de las últimas. Siete manuscritos publicados (dos editoriales, la colaboración especial, dos notas clínicas, un original y una carta) no estuvieron firmados por ninguna mujer; en todos los casos el firmante era un único autor, excepto en el original, que eran dos.

Como es habitual, la mayoría de los artículos publicados provino de España (83%), con Navarra (31%) como comunidad más representada. Más de la mitad de los artículos proveniente de otras comunidades correspondió a Aragón (10%), Andalucía y Murcia (8% cada una de ellas).

**Figura 2:**
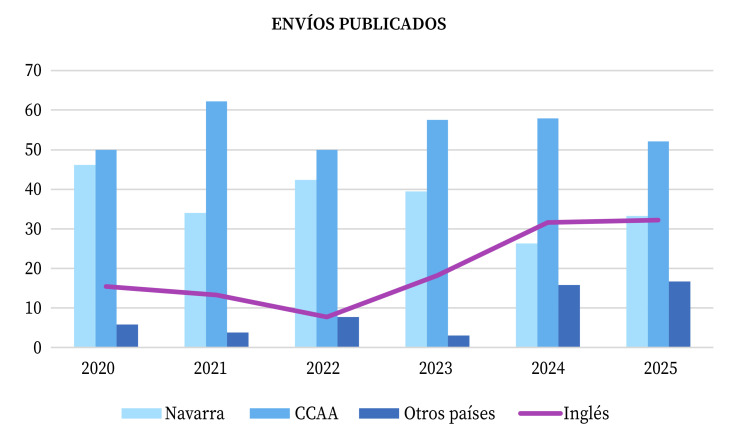
Procedencia geográfica de los envíos publicados entre 2020 y 2025 en la revista Anales del Sistema Sanitario de Navarra, y porcentaje de los mismos en inglés

Las contribuciones internacionales se mantuvieron en 2025 (17%); los artículos con autorías de América del Sur/Central representaron casi dos tercios frente al tercio de los procedentes de Asia (10,4% y 6,25% respecto del total de publicados).

El 32% de los artículos se publicó en inglés, porcentaje casi idéntico al de 2024.

## ÁREAS DE ESTUDIO DE LAS PUBLICACIONES

La pandemia de COVID-19 tuvo un impacto muy importante en nuestro país y sigue siendo fuente de estudios que generan conocimientos útiles para la práctica clínica. Un estudio de cohortes en atención primaria documentó que hasta el 32% de los pacientes presentaba síntomas de COVID persistente, principalmente astenia y anosmia/disgeusia, dos años después de la primoinfección^2^. La persistencia de algún síntoma se asociaba con la gravedad inicial y la reinfección, afectando negativamente a la calidad de vida. Otro estudio internacional señalaba que también la preexistencia de factores de riesgo cardiovascular comportaba una mayor probabilidad de secuelas a largo plazo en adultos mayores de 60 años. Prevenir y controlar estos factores es fundamental para reducir los efectos a largo plazo de la COVID-19^3^.

Se han realizado contribuciones muy notables en el área de las enfermedades infecciosas. Un programa novedoso de diagnóstico y tratamiento precoz del VIH en urgencias de un hospital, centrado en pacientes que reunían características como neumonía adquirida o infección de transmisión sexual, detectó infección por el VIH en el 3,6% de los pacientes^4^. Este hallazgo es especialmente relevante porque eran personas que no acccedían regularmente al sistema sanitario. Por tanto, la implementación del programa de diagnóstico de VIH en servicios de urgencia puede ser una herramienta eficaz para la detección precoz de la infección en este tipo de pacientes. También en el ámbito de las enfermedades transmisibles, se estudió la efectividad de la inmunización con nirsevimab en neonatos para prevenir hospitalizaciones por virus respiratorio sincitial (VRS) durante dos temporadas en Navarra^5^**.** La inmunoprofilaxis con nirsevimab resultó efectiva para prevenir hospitalizaciones por VRS y alivió la sobrecarga de ingresos pediátricos. Sin embargo, ante el riego de casos en inmunizados, la inmunoprofilaxis debe complementarse con otras medidas preventivas.

La salud laboral también ha sido objeto de atención en otros artículos. En un estudio en la comunidad foral de Navarra se estudió la intención de abandonar la profesión de enfermera durante la sexta ola de la pandemia por COVID-19^6^. La intención de abandono se asoció con niveles moderados de ansiedad y estrés postraumático, y con menor experiencia profesional. Los autores recomendaron mejorar las condiciones laborales y promover el bienestar mental, especialmente en contextos de crisis sanitaria. En otro estudio realizado en un hospital de tercer nivel, se analizó la situación emocional, motivación y satisfacción del personal de enfermería, y se constataron altos niveles de desgaste profesional^7^. El estudio recomendó reforzar el liderazgo de los supervisores y diseñar un programa para mejorar la relación entre los objetivos institucionales y necesidades percibidas por el equipo asistencial.

La violencia de género ha emergido como uno de los problemas de salud más relevantes, y los sistemas de salud son clave para detectar y abordar este problema. Un estudio evaluó la efectividad de un programa formativo para mejorar los conocimientos, habilidades y actitudes del personal sanitario de urgencias y emergencias frente a la violencia de género mediante cursos breves y en línea, utilizando recursos innovadores como vídeos-problema dramatizados^8^.

Los aspectos relacionados con la organización y la práctica clínica se han abordado desde diferentes perspectivas. Un estudio revisó las intervenciones para mejorar la experiencia de familias y acompañantes durante la espera quirúrgica, y optimizar el apoyo emocional para la mejora de la experiencia y bienestar en este contexto^9^. La calidad de los registros, especialmente en el área quirúrgica, es un elemento clave para la seguridad de los pacientes y una investigación detectó omisiones relevantes con potenciales consecuencias sobre la seguridad del paciente^10^. El dolor musculoesquelético es un problema frecuente en práctica clínica que afecta la calidad de vida. Un estudio investigó, en los estudiantes de ciencias de la salud, la prevalencia de dolor musculoesquelético y su efecto negativo en la salud mental^11^.

El deterioro cognitivo subjetivo y leve afecta a una proporción elevada de la población adulta. Mediante un ensayo controlado aleatorizado se compararon dos intervenciones (estimulación cognitiva computarizada y actividades de ocio estimulantes) y se constató que podían mejorar de forma complementaria los síntomas de ansiedad y depresión, y la cognición global, en personas con deterioro cognitivo leve^12^.

El cribado de cáncer colorrectal es una intervención de salud pública con una efectividad contrastada para reducir la mortalidad y los programas tienen un margen de mejora importante para aumentar su cobertura. Un estudio valoró el impacto de la pandemia de COVID-19 en la cobertura, la diferente participación de mujeres y hombres y el porcentaje de cambio anual relacionado con el objetivo de participación de la Estrategia en Cáncer española^13^.

## RETOS Y LÍNEAS DE TRABAJO

Los resultados de Anales del Sistema Sanitario de Navarra en 2025 recogen parte de las investigaciones sobre problemas relevantes que tiene que abordar el sistema sanitario, y aportan conocimiento sobre aspectos susceptibles de mejora en temas importantes como el dolor muculoesquelético, las experiencias de familiares en zonas de espera en el área quirúrgica, la detección de la infección de VIH en áreas de urgencia o las intervenciones recientes y novedosas con la inmunoprofilaxis con nirsevimab para reducir las bronquiolitis en lactantes. También las notas clínicas han recogido de forma breve y práctica aspectos que fomentan el intercambio de ideas y opiniones entre profesionales. Estos resultados nos animan a seguir publicando estudios que aporten conocimiento significativo para la práctica profesional y la mejora de la salud de la población.

Las presiones por publicar han seguido en aumento y la proliferación de proyectos, artículos y plataformas para publicar no ha dejado de crecer en los últimos meses. En este escenario, los informes de los revisores que aportan su juicio de expertos sobre el significado y la calidad de las potenciales publicaciones son cruciales para que los editores prioricen los artículos que aportarán más valor al sistema sanitario. En este editorial también queremos reconocer la generosidad de todas las personas que han regalado parte de su tiempo y *expertise* en la evaluación de manuscritos.

La difusión masiva de la inteligencia artificial generativa en noviembre de 2022 ha generado expectativas de rapidez y mejora en la producción editorial, pero también inquietud y preocupación por su uso inapropiado. La reciente actualización de la guía del Comité Internacional de editores de Revista Médicas^14^ recomienda no asignar categoría de autor a las herramientas de IA, y a los autores les aconseja usarlas solo en apoyo a su trabajo, pero nunca como sustitutos de su papel. Los autores deberían declarar el uso de las IA detallando la plataforma y el uso realizado. Los revisores tampoco deben usar las IA para elaborar sus juicios como expertos porque podrían vulnerar la obligación de confidencialidad de su labor. Los potenciales sesgos, el uso de referencias incorrectas e incluso plagios son objeto de especial atención en estos momentos, pero creemos que la declaración del uso de la IA no debe ser estigmatizada si se usa de forma transparente y trazable.

En este escenario de continuos cambios, Anales del Sistema Sanitario de Navarra quiere seguir en la senda de aportar conocimiento útil y práctico para el conjunto del sistema sanitario a través de publicaciones éticas, transparentes y con calidad contrastada.
